# Experimental Evaluation of Industrial Mushroom Waste Substrate Using Hybrid Mechanism of Vermicomposting and Effective Microorganisms

**DOI:** 10.3390/ma15092963

**Published:** 2022-04-19

**Authors:** Khalid Ansari, Shantanu Khandeshwar, Charuta Waghmare, Hassan Mehboob, Tripti Gupta, Avinash N. Shrikhande, Mohamed Abbas

**Affiliations:** 1 Department of Civil Engineering, Yeshwantrao Chavan College of Engineering, Nagpur 441110, India; khandeshwar333@gmail.com (S.K.); charutawaghmare@gmail.com (C.W.); 2 Department of Engineering Management, College of Engineering, Prince Sultan University, Riyadh 11586, Saudi Arabia; hmehboob@psu.edu.sa; 3 Department of Civil Engineering, Shri Ramdeobaba College of Engineering and Management, Nagpur 440013, India; guptatb@rknec.edu; 4 Department of Civil Engineering, Kavikulguru Institute of Technology and Science, Ramtek 441106, India; dranskitsr@gmail.com; 5 Electrical Engineering Department, College of Engineering, King Khalid University, Abha 61421, Saudi Arabia; mabas@kku.edu.sa; 6 Computers and Communications Department, College of Engineering, Delta University for Science and Technology, Gamasa 35712, Egypt

**Keywords:** mushroom waste, vermicomposting, effective microorganisms, XRD method, FTIR method

## Abstract

Mushroom waste substrates are highly resistant lignocellulosic wastes that are commercially produced by industries after harvesting. These wastes produce large environmental challenges regarding disposal and, thus, require treatment facilities. In the present article, the effect of *Eisenia-fetida*-based vermicomposting and an effective microorganism solution on the mushroom waste substrate were investigated using four different composting mixtures: mushroom waste [MW] substrate composting with effective microorganisms [MW+EM], raw mushroom waste [RWM] substrate composting with effective microorganisms [RMW+EM], mushroom waste substrate composting with vermicomposting and effective microorganisms [MW+V+EM], and raw mushroom waste substrate composting with vermicomposting and effective microorganisms [RWM+V+EM]. This article discusses the structural and physiochemical changes at four samples for 45 days (almost six weeks) of composting. The physical and chemical parameters were monitored during composting and provided information on the duration of the process. The results indicated pH (7.2~8), NPK value (0.9~1.8), and C:N ratio <14, and heavy metals exhibited a decreasing trend in later stages for all sets of compost materials and showed the maturity level. FTIR spectra revealed that all four samples included peaks for the -OH (hydroxy group) ranging from 3780 to 3500 cm^−1^ and a ridge indicating the C=C (alkenyl bond) ranging from 1650 to 1620 cm^−1^ in compost. The X-ray diffraction spectrum clearly shows how earthworms and microbes break down molecules into cellulose compounds, and the average crystallinity size using Scherrer’s equation was found to be between 69.82 and 93.13 nm. Based on the experimental analysis, [RWM+V+EM] accelerated the breakdown of organic matter and showed improvement compared with other composts in compostable materials, thus, emphasizing the critical nature of long-term mushroom waste management and treatment.

## 1. Introduction

Mushroom farming is a green enterprise since it recycles the waste from farms, animals, breweries, and other sources while producing fruit bodies with unique nutritional and medicinal properties [1] Every year, 6–7% more global mushroom production is grown in over 100 countries with high-tech mechanization and automation, particularly in wealthy China (36%) as well as European (18%) and North American countries (7%). Currently, mushroom farming in India is growing 30–40% annually [2]. Wheat straw, soybean straw or paddy, and other agricultural wastes are valuable residues of edible mushroom production described in Table 1. Mushroom substrates generate almost 1–2 tonnes of waste for every tonne of mushrooms collected [3] and are generally disposed of in landfills, which causes eutrophication of the surface water basins through nutrient leaching or composted after mushroom harvest [4] as well as commonly disposed of through open burning, producing airborne hazards [5,6,7,8,9,10,11,12,13,14].

As a result, mushroom waste contains high concentrations of salts and organics, which cause numerous environmental issues and are difficult to handle and dispose of using traditional disposal and burning methods, which is again improper and unsupervised. In recent years, ecological restrictions have increased the pressure on mushroom producers, underlining the essential need for a more suitable means of disposing of waste, which includes direct application to the soil as a bioremediation agent and animal and fish feed [3,4]. On the other hand, composting can be a suitable technology that is a cost-effective and environmentally friendly option for disposing of mushroom waste [6]. It is a biological process (agricultural waste disinfectant turned into a corresponding and usable flora matter) that occurs in the presence of sufficient oxygen, humidity, and temperature [7]. 

In composting, microorganisms generate heat, and a solid substrate is converted into less carbon and nitrogen. This method is time-consuming. For different quality organic wastes, frequently aeration is required, which depends on the ingredients [4,5,6,7,8]. Earthworm-based composting (vermicomposting) can decompose organic waste to produce odourless humus-like substances that are beneficial to the environment, according to the scientific literature in recent years [7,8,9,10]. Vermicomposting is a key biotechnological composting technology in which various earthworms are adapted to improve the waste conversion process [11,12,13,14], combining an advanced microbe technology with a vermicomposting approach, such as effective microorganism technology, can reduce the time required and shows the stability and maturity of the product. 

Effective microorganism (EM) technology is a method for the natural agricultural protection of live microorganism communities isolated from naturally rich soils and used as a by-product to improve the earth’s biodiversity, resulting in increased agricultural yields. The most crucial effective microorganisms are *Lactobacillus plantarum*, *Lactobacillus casei*, *Streptococcus lactis, Rhodopseudomonas palustrus, Rhodobacter spaeroides*, *Streptomyces albur*, *Mucorhiemalis,* and *Aspergillus oryzae* [6]. These include lactic acid bacteria, which is a powerful sterilizing ingredient that suppresses pathogenic germs and accelerates the degradation of organic material [9,10]. 

With the rapid generation of mushroom waste, it can be valorized for agricultural applications in terms of compost, which in turn increases the soil mineral nitrogen, specifically nitrate (NO_3_^−^) and ammonia (NH_3_), making it an excellent soil conditioner and natural soil insecticide [21,22,23,24,25,26,27,28,29,30,31,32,33,34,35,36,37,38,39]. Researchers have focused on new and alternative energy resources derived from the mushroom waste substrate, which includes vast amounts of lignocellulosic components, such as cellulose, hemicellulose, and lignin that can be converted to bio-oils using modern heating technologies [39]. 

Thus, the objective of this research is to evaluate the composting process by examining the transformation of mushroom waste and raw mushroom waste substrates into mature and stable compost by using the effective microorganism alone and hybrid mechanism of vermicomposting and effective microorganisms concerning temporal and physicochemical characteristics associated with the structural behaviour during compost development.

## 2. Materials and Methods

### 2.1. Mushroom Waste Substrates Collection

Mushroom waste (MW) substrates are the waste materials collected from the mushroom industry at nearly two months after harvesting, and they are dried and stored for ten days. In contrast, raw mushroom waste substrates (RMW) are the materials harvested at one to two days that are dried and stored for ten days. For the partial decomposition, both wastes were kept at an ambient temperature of (25~30 °C) with relative humidity (60~90%), consisting of a mixture of wheat straw (*Agaricus bisporus*) and soybean straw (*Pleurotus* sp.) ranging from 1.0 to 5.0 cm, which was brought in a plastic container from Balaji Farms Pvt. Ltd. in Khond Hali, Wardha, Nagpur, India. Prior to the experimental trial, both waste products were sliced and sieved to a particle size of 0.6~0.9 mm to increase the material’s homogeneity and digestion by earthworms. The initial chemical characteristics of the materials are presented in Table 2.

### 2.2. Activation of Effective Microorganisms (EM)

The EM solution is activated using the Bokashi method (EM-1^®^ solution, jaggery, and water). Jaggery is a lump of non-centrifugal cane sugar that has been consumed in India and Southeast Asia for centuries, containing up to 50% sucrose, 20% invert sugar, and 20% moisture, and the remainder consisting of different insoluble substances, such as wood ash, proteins, and bagasse fibres [4,5,6]. To activate the EM solution before usage, a dormant EM-1^®^ solution, roughly 1000 mL, was mixed in an airtight container with 2 kg jaggery and 20 Liters of distilled water. It was then stored at room temperature for 8 to 10 days, away from direct sunlight, and absorbed actinomycetes during activation at the top of the surface with a pleasant smell and a pH below 3.3~3.5. [15].

### 2.3. Earthworms Culture

*Eisenia-fetida* (earthworms’ species) were obtained from Sath Company, Narendra Nagar, Nagpur, India. Earthworms were brought in with a quantity of 1.5 kg; weight between 0.5 and 1 g, their length between 2 and 4 cm, and their breadth between 1 and 2 mm. They were maintained using 3 kg cow manure and 1 kg of soil as a culture medium for 15 days in a container with mechanical aeration, with a moisture content of 55% to 65%, and a pH for earthworm survival ranging between 7.0 and 7.6. The C:N ratio was maintained at less than 20, to provide favourable environmental conditions for earthworms.

### 2.4. Experimental Set Up

**Figure 1:**
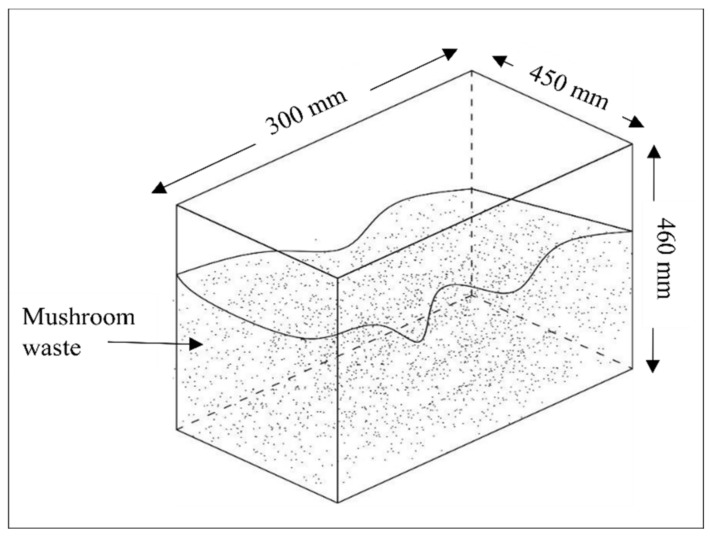
Illustrated diagram of a rectangular glass jar.

Four experiments were conducted under aerobic conditions, using rectangle glass jars (with dimensions of 300 × 450 × 560 mm) and a top opening with a 1 cm small orifice opening on each side of the jar for drainage as shown in Figure 1.

#### 2.4.1. Effective Microorganism Design

**Figure 2:**
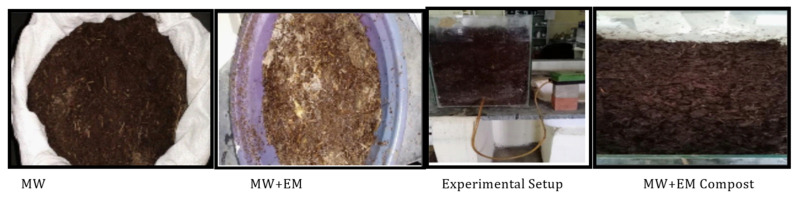
Set up 1 shows the images of mushroom waste with EM [MW+EM].

**Figure 3:**
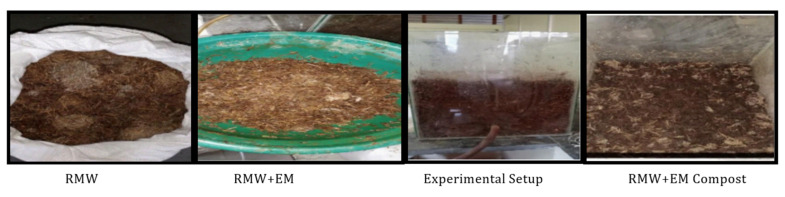
Set up 2 shows the images of raw mushroom waste with EM [RWM+EM].

Figure 2 shows set-up 1, which consisted of 10 kg of mushroom waste [MW] substrate on a dry weight basis in a glass jar and was served in four layers at a depth of 100 mm, with 240 mL (60 mL on each layer) of activated EM solution and 4000 mL (1000 mL on each layer) of water spread over the layers. After every three days, the container of waste substrates was opened for aeration and to maintain its moisture content of approximately 50–60% by periodically sprinkling an adequate quantity of water [6]. The same replicates were prepared with raw mushroom waste in set-up 2, as shown in Figure 3.

#### 2.4.2. Vermibed Design

**Figure 4:**
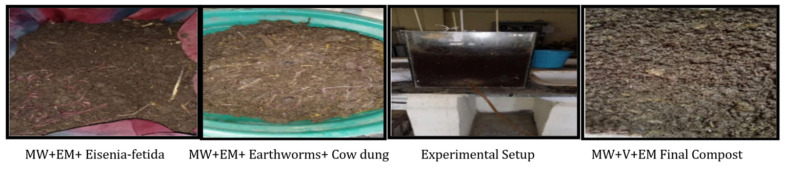
Set up 3 shows the images of mushroom waste with vermicomposting and EM [MW+V+EM].

**Figure 5:**
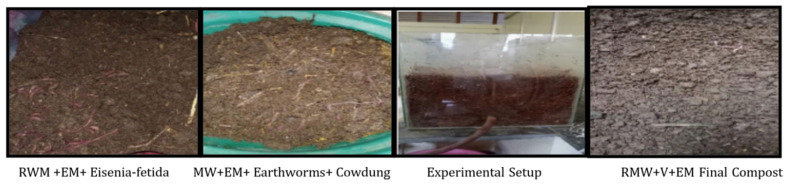
Set up 4 shows the images of raw mushroom waste with vermicomposting EM [RMW+V+EM].

Figure 4 shows set-up 3, which consisted of 10 kg of mushroom waste [MW] substrate on a dry weight basis in a glass jar and was served in four layers at a depth of 100 mm, with 240 mL (60 mL on each layer) of activated EM solution and 4000 mL (1000 mL on each layer) of water spread over the layers. 40 healthy earthworms (10 in each layer), approximately 4–6 cm in length and (1.5–3) g in weight, were introduced after 15 days of partial decomposition of wastes substrates, and 3 kg of cow dung was mixed in a container. The container of waste substrates was aerated every three days, and its moisture content was maintained at around 50 to 60% by spraying a suitable amount of water regularly [6]. The same replicates were created in set-up 4 with raw mushroom waste, as indicated in Figure 5.

The experiments lasted for 6 weeks with the completion of compost of each set of an experimental container, and the examined samples were dried in an oven at 60° C for 48 h, pulverized in a stainless-steel mixer, and stored in sterilized plastic containers [7]. The glass jar was covered with jute cotton cloths to prevent moisture loss and direct sunlight. The details of each set (1 to 4) from raw waste to compost are shown in Figure 2, Figure 3, Figure 4 and Figure 5, respectively.

### 2.5. Physico-Chemical Analysis

It is critical to assess mixed and homogeneous compost’s quality, maturity, and nutrient content before deciding on its potential uses. Based on the experimental analysis, each compost (50 g) sample was taken every week and was homogenously mixed for analyses of pH, temperature, odour, colour, C:N ratio, and changes in humic acid. The preparation of pH was done with 1 g of compost and 5 mL of distilled water and checked in pH digital electrode meter after every three days [11]; the temperature was checked regularly at an interval of three days with the thermometer at a depth of 60% from the top at three different zones of the jar, colour and odour were observed every three days interval visually and smelling [4]. 

The Hach TOC Bio-Tector B3500 C (HACH, Loveland, CO, USA) was used to assess The Total Organic Carbon (TOC) concentrations in pulverized dry materials. The micro-kjeldahl technique was used to determine The Total Nitrogen (TN). The C:N ratio was calculated as TOC/TN. The concentration of NPK was determined using Spectroquant test kits using soluble potassium (K_2_O), and soluble phosphate (P_2_O_5_) were analysed photometrically. Solution reagents were put in test kits [11], and heavy metals like Fe, Cu, and Zn were determined using standard procedures using atomic absorption spectroscopy (SYSTRONICS, Ahmedabad, India) [3,11].

The structure of humic acids and structural changes induced in a crystalline composting material was characterized by FTIR and XRD analysis performed at the Chemistry Department of Nagpur University (Nagpur, India). Samples from each set of composting after 6 weeks were oven-dried and coarsely pulverized for X-ray diffraction analysis using an X-ray diffractometer (Philips PRO model) (Philips, U.K) equipped with a copper length anticathode and operated with radiations (λ = 1.5406 A°) with data capturing angles of (2θ) [16].

The Crystallinity size (D in nm) was calculated by Scherrar Equation [22].

D = [(K λ)/(β.cosθ)](1)

where D = crystallites average size (nm),

K = Scherrer’s constant (0.94 for spherical crystals shape),

λ = wavelength of radiation (0.1546 nm),

β = FWHM (in radian) full width at half maximum intensity,

θ = Bragg angle at peak position = 2θ (in radian)

FTIR spectra were identified using a (Bruker Vertex 70 spectrometer model) (Bruker, UK) with OPUS 6.5 software for data manipulation and statistical analysis equipped with attenuated total reflection (ATR). A 5 mg of each sets samples were oven-dried and grounded with a spectroscopic grade of KBr in (1:100) ratio with scanning range of 4000–400 cm^−1^ at a rate of 0.5 cm/s [3] to ascertain the behavior of humic acids and visualize both their infinitesimal structure and their microscopic environment.

### 2.6. Statistical Analysis

The significant difference between the initial and final compost results for the parameters was investigated using one-way ANOVA. A Tukey’s *t*-test was used to analyse the data, and all values are reported as the mean ± SE. For the tests, the probability thresholds utilized for statistical significance were *p* ≤ 0.05.

## 3. Results and Discussion

### 3.1. Temperature

Temperature is a very important environmental factor that specifies the metabolic intensity and organic waste changes during microorganism activity [17,22]. Each set increased rapidly and gradually over the first few days, i.e., during the first and second weeks of the composting process, peaking between 42 and 55 °C, indicating the development and metabolic activity of the microbial community within the compost quantity, and then decreasing to an ambient temperature of (25–33) °C. 

**Figure 6:**
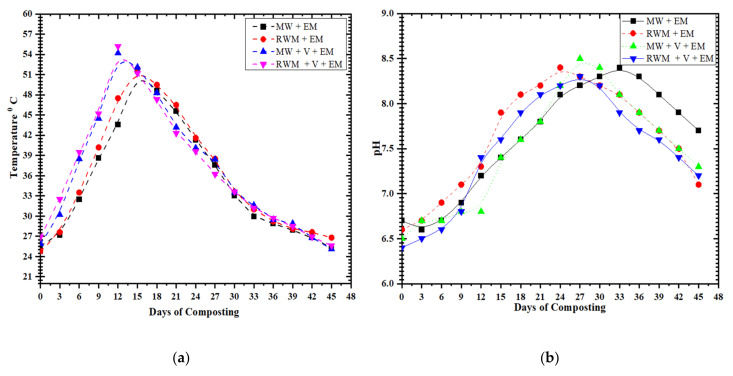
Variation of temperature (**a**) and pH (**b**) changes during composting days.

This demonstrates that the [MW+V+EM] (56.2 °C) and [RMW+V+EM] (58.3 °C) samples reached the thermophilic stage (>45 °C) and lasted for 12–14 days, in comparison to the [MW+EM] (50 °C) and [RMW+EM] (52 °C) samples, which reached the thermophilic stage (>45 °C) and lasted for 8–10 days, as shown in Figure 6a, which shows that the EM maintained the minimum requirement of thermophilic stages in all the sets and due to aeration effects, higher biodegradation activity is possible during the loading period [8] in the feedstock via the heat generated by the microorganism population’s respites, and substrate breakdown is possible [1,2,3]. Again, it was seen that the temperatures of all the sets began to drop at the beginning of the third week, suggesting the maturity of the organic matter as demonstrated by the mass reduction in compost volume and odour emission as well [15].

### 3.2. pH and Odour

As seen in Figure 6b, the pH increase in all four sets of samples from the acidic original compost during the initial days and turned to neutral and alkaline (pH > 8), which results in the microbial activity converting organic acid to CO_2_, suggesting that the organic matter was stable [10,26], and this had an unusual odour. According to researchers, the appropriate scale for high-quality compost is between pH 6 and 8.5 [25]. Composts made from vermibed waste, such as [MW+V+EM] and [RMW+V+EM], gradually lost pH in comparison to [MW+EM] and [RMW+EM], owing to CO_2_ and the loss of organic acids due to ammonia volatilization during composting [23]. Additionally, it was observed that the pH values of all substrates decreased after the fourth week of composting, which could be due to the biochemical properties of organic acids resulting in an increase in the microbial population, which results in increased manure development, and the pH slightly approaches neutrality [38].

Composting generated odour in all four sets, which indicates the presence of organic waste and other metabolic products produced entirely aerobically and anaerobically [1,31], thereby, increasing and upgrading the bacterial population responsible for decomposition in organic matter during composting, as well as providing a beneficial environment [24,25,26,27,28]. In addition, we observed that the unpleasant smell associated with the composting decreased over time due to the degradation of the material [27,29]. In addition, [MW+V+EM] and [RMW+V+EM] produced an earthy smell in a shorter time due to the capability of earthworms for bioconversions of the substrate to compost compared with [MW+EM] and [RWM+EM].

### 3.3. C:N Ratio

**Figure 7:**
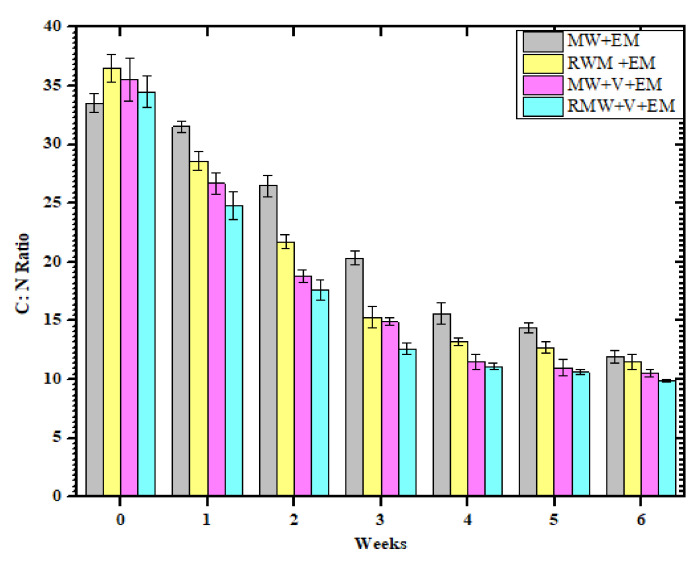
Variation of the C:N ratio within different weeks of [MW+EM], [RMW+EM] [MW+V+EM] and [RMW+V+EM]. Error bars represent standard errors for six samples.

As illustrated in Figure 7, the carbon to nitrogen ratio had the most significant effect on the composting maturity efficiency [30,31]. According to present research, the carbon-to-nitrogen ratio final reductions in each set of composters with 77% in RMW+V+EM were slightly greater than 69% in MW+V+EM, which is significantly more than in the composters consisting of 59% RWM+EM and 55% MW+EM, which may be due to organic conditions composed of inorganic metabolizable components [7]. 

Within the first four weeks, the carbon-to-nitrogen ratio of all EM-treated samples decreased significantly as carbon was primarily evaluated as carbon dioxide, while nitrogen was lost via volatilization [35] and showed maturity and phytotoxic by the competition of 6 weeks. According to experts [32], the C:N balance should be at least 20 for optimal quality and mature compost. The C:N ratio did not differ more between composts with and without earthworms (*Eisenia-fetida*), indicating that the degradation rate was similar in all situations.

### 3.4. Heavy Metals

**Figure 8:**
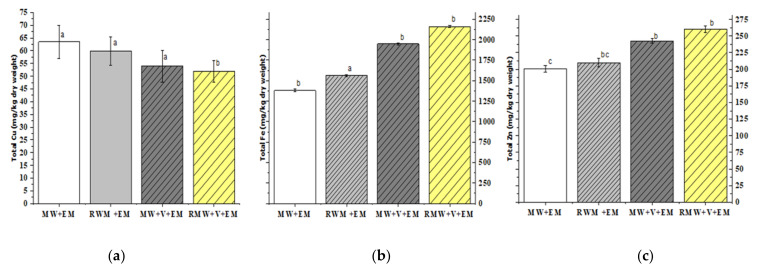
The total concentrations of Cu (**a**), Fe (**b**), and Zn (**c**) of [MW+EM], [RMW+EM], [MW+V+EM], and [RMW+V+EM]. Error bars represent the standard errors for six samples. Columns followed by the same letter do not differ significantly (ANOVA; Tukey’s test, *p* < 0.05).

After the end of the composting process, the vermicomposting sets of containers showed a significant (*p* < 0.05) increase in the metal content, as shown in Figure 8a–c. The accumulation of heavy metals, such as iron (Fe) and manganese (Zn), in [RWM+V+EM] and [MW+V+EM] (*p* < 0.05) was due to metal bioaccumulation in earthworm tissues, which accelerates the decomposition rate due to a higher temperature of 54 °C. The additionally effective microorganisms were responsible for the breakdown of organic waste and enhanced humification process indicating that the compost had matured [6,37]. The same was observed for [MW+EM] and [RWM+EM] with a slow pace of increase, whereas copper (Cu) (*p* < 0.05) decreased proportionately with time for all sets of samples displaying that the available waste particles were converted into useless small particles as a result of earthworm and microorganism activity [37]. 

Indirectly, these findings support that heavy metal removals, such as (Cu and Zn) use of show a maturity limit of compost sets for in EU countries {for Cu (mg/kg), the limit range is 70~600 and for Zn (mg/kg), the limit range is 210~4000} and for the USA (for Cu (mg/kg), the limit range is 1500 and for Zn (mg/kg), the limit range is 2800) [39]. Heavy metals, such as Fe, Zn, and Cu are micronutrients that are critical for plant growth and rapidly rise and fall during the composting process, thus, indicating the compost’s development.

### 3.5. Evaluation of NPK

**Figure 9:**
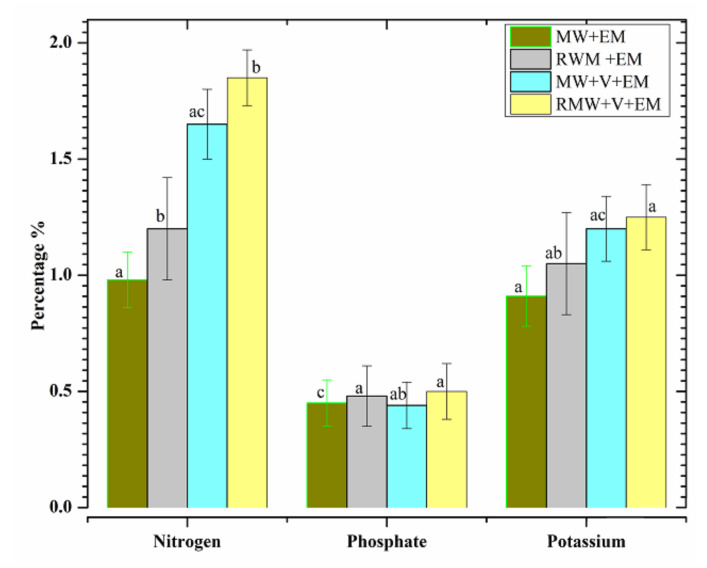
The total concentrations of the NPK values of [MW+EM], [RMW+EM], [MW+V+EM], and [RMW+V+EM]. Error bars represent standard errors for six samples. Columns followed by the same letter do not differ significantly (ANOVA; Tukey’s test, *p* < 0.05).

Other key components, nitrogen, phosphorus, and potassium (NPK), as illustrated in Figure 9, revealed that adding EM solution to the compost samples boosted their value. The nitrogen contents of [MW+EM] and [RMW+EM] were significantly higher in this study than in [MW+V+EM] and [RMW+V+EM], owing to the usage of nitrogen by microbes to create cells that operate as nitrogen-fixing biological organisms throughout the compost growth process. The total nitrogen concentrations should generally range between 1% and 3% by dry weight [4]. The statistics (*p* < 0.05) indicate that EM and vermicomposting had a good effect, with desired values ranging from 0.9% to 1.8%for each of the four samples.

Phosphorus is a vital nutrient, and the available phosphate is fine-tuned by Fe^3+^ and Al^3+^ ions in an acidic environment. The results suggest that the phosphorous nutrient statistic (*p* < 0.05) decreased somewhat with or without the input of earthworms, with a range of around 0.43% to 0.5%. Potassium is required to form proteins and carbohydrates and to regulate di-hydrogen monoxide levels during culture. The findings of potassium levels in [MW+EM] and [RWM+EM] indicate that the compost was substantially more potent than [MW+V+EM] and [RWM+V+EM], as earthworms added microbial-mediated nutrient mineralization to the final product. Overall, the NPK values suggest that earthworms and efficient microbial activities are necessary for optimal nutrient absorption.

### 3.6. FTIR

**Figure 10:**
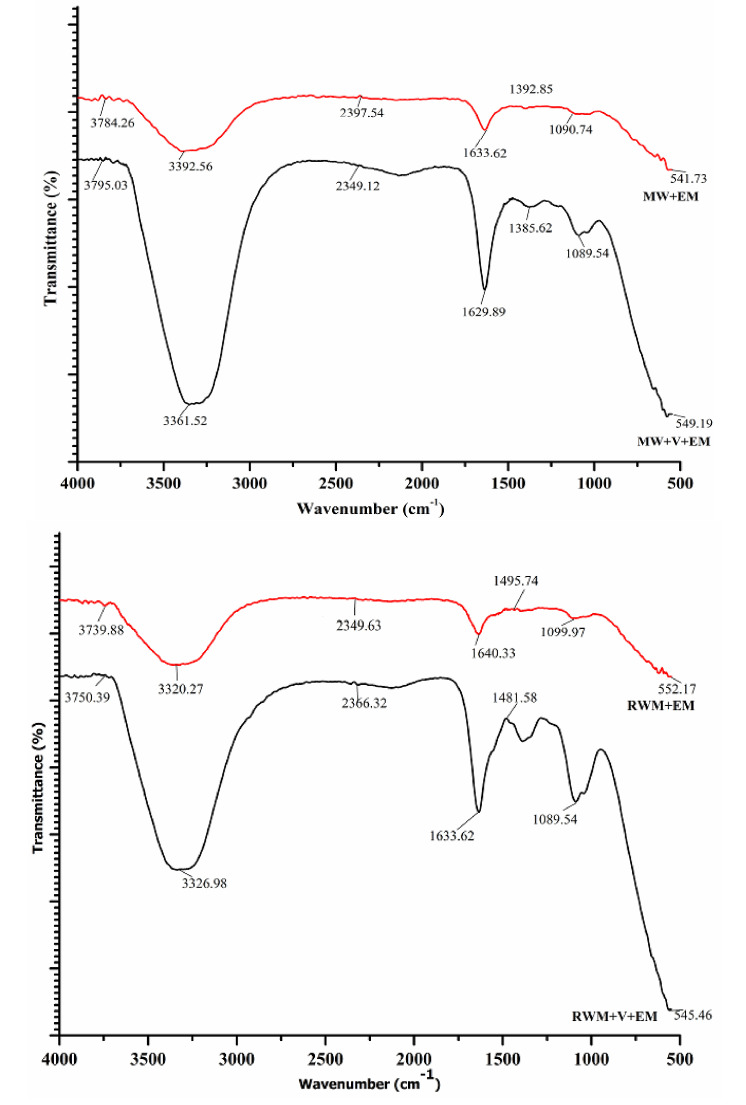
Variation of FTIR of the samples: [RWM +EM], [MW+EM], [MW+ V+EM], and [RWM+V+EM].

The FTIR analysis of the mushroom waste substrate compost samples was used to examine the existence or absence of a functional group, as well as the degradation or stabilization process. The FTIR study indicated changes in the material characteristics for [MW+EM], [RWM+EM], [MW+V+EM], and [RWM+V+EM] to demonstrate the variance in the IR spectroscopic bands, as seen in Figure 10. The ranges [MW+EM] at 3392.56 cm^−1^, [RMW+EM] at 3320.27 cm^−1^, [MW+V+EM] at 3361.52 cm^−1^, and [RMW+V+EM] at 3326.98 cm^−1^ reflect the hydroxy group, H-bonded OH stretch, and robust, wide absorption intensity, respectively. 

Due to the inadequate absorption intensity of band 2397.54 cm^−1^ in [MW+EM] and 2349.12 cm^−1^ in [MW+V+EM], the existence of a terminal alkyne (monosubstituted) molecule was detected. The alkenyl C=C stretch resulted in a medium absorption rate (C=C in the aromatic region) at 1633.62 cm^−1^, 1629.89 cm^−1^, 1640.33 cm^−1^, and 1633.62 cm^−1^ in [MW+EM], [MW+V+EM], [RMW+EM], and [RMW+V+EM], respectively. The bands at 1090.74 cm^−1^ in [MW+EM], 1099.97 cm^−1^ in [RMW+EM], and 1089.54 cm^−1^ in [MW+V+EM] and 1089.64 cm^−1^ in [RMW+V+EM] correspond to a secondary alcohol—a C-O stretch molecule with a dynamic molecular structure. 

The bands at 541.73 cm^−1^, 549.19 cm^−1^, 552.17 cm^−1^, and 545.46 cm^−1^ in [MW+EM], [MW+V+EM], [RMW+EM], and [RMW+V+EM], respectively, indicate Aliphatic Bromo compounds—a C-Br stretch with a high absorption intensity [15,28]. The presence of an aromatic region indicates that stable compounds are being formed. Ultimately, these results reveal that the increasing lowering of peaks over the composting days reflects the modifications presented in the structural components via the activity of effective microorganisms [28].

### 3.7. X-ray Diffraction Analysis

**Figure 11:**
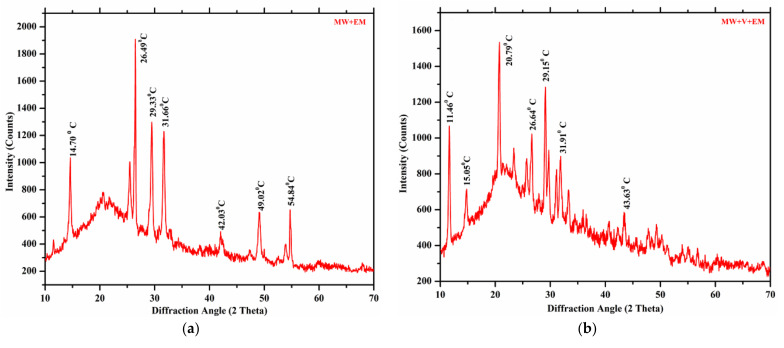

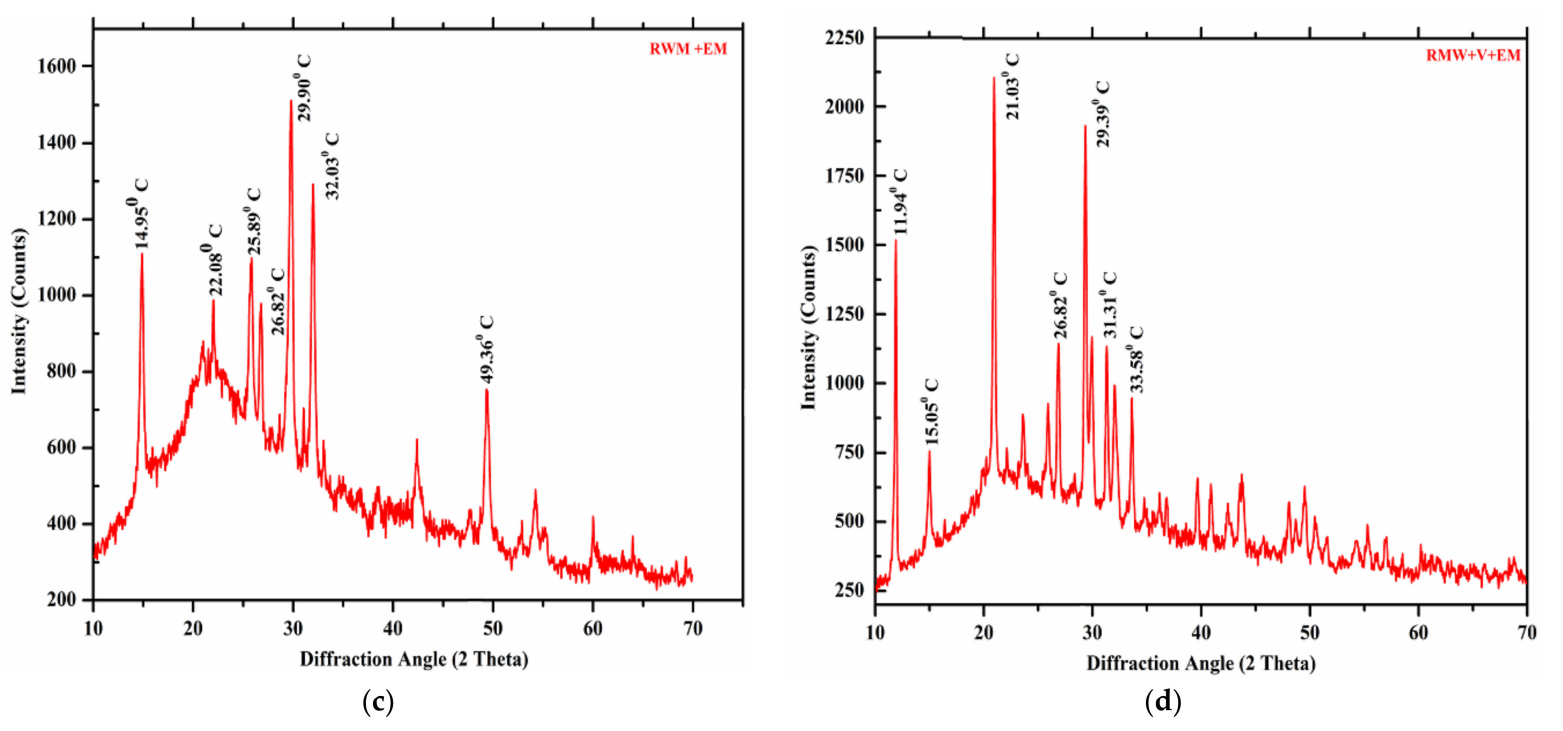
XRD analysis of the sample (**a**) [MW+EM], (**b**) [MW+V + EM], (**c**) [RWM+EM], and (**d**) [RMW+V+ EM].

X-ray diffraction is a supplementary approach for deciphering a compound’s structural features. Figure 11 shows changes in the peak heights for all XRD signals representing crystal planes from 10 to 70 diffraction angles, which illustrates the spectroscopy of various mushroom waste substrates composting samples, such as (a), (b), (c), and (d) collected after completion of six weeks, indicating a decrease in the number of peaks levels due to an increase in decomposition processes and indicating the formation of ready manure. 

The findings showed that [RWM+V+EM] and [MW+V+EM] have acute peaks appearing at 2θ of 21.03 and 20.79, respectively, in the early stages, indicating a decrease in the C:N ratio and thus a reduction of cellulose compounds, which results in the destruction of hydrogen bonds and is easily destroyed [28], whereas [RMW+EM] and [MW+EM] had sharp peaks appearing at 2θ of 29.90 and 26.49, respectively, related to the crystalline nature of cellulose. The X-ray diffraction technique used in Equation (1), to calculate the cross-sectional dimension of cellulose crystallites, and Scherrer’s equation is useful for approximating the crystallite size. 

The data in Table 3 demonstrate that, according to the results of the FWHM measurement, the crystallite size of [MW+EM], [RWM+EM], [MW+V+EM], and [RWM+V+EM] are 93.13, 86.52, 72.43, and 69.82 nm, respectively. It has been found that crystallinity plays a significant role in the mechanical and physical properties, which results in the strength and stiffness of the cellulose fibres [35,36,37,38,39]. Lastly, the XRD spectrum indicates that the particle size of mushroom waste substrates reduced during the decay phase of the compost material. In a nutshell, the XRD data showed that all composting samples generated cellulose, which was attributed to the presence of earthworms and microbiological activity [40,41,42,43,44,45,46,47,48,49,50].

### 3.8. Duration and C:N Ratio: Comparison with Other Studies

Table 4 shows the results of the previous and present study’s treatment of mushroom waste substrate compared to other vermicomposting using earthworm species. The present study demonstrates the effectiveness of duration, the C:N ratio, and the rapid development of mature and stable compost, which opens a new method that can reduce the volume of cumulative waste in the environment [51,52,53,54,55,56,57].

## 4. Conclusions

A composting experiment was conducted on mushroom waste substrate and raw mushroom waste substrate with the application of effective microorganisms and a hybrid method of vermicomposting and effective microorganisms over 6 weeks. The final four experimental set-ups resulted in nutrient-rich compost that improved in odour and colour and showed maturity and stability. 

The compost with effective microorganisms and vermibed demonstrated an accelerated increase in N, P, and K values as well as a decrease in the C:N ratio that was slightly higher than the effective microorganisms alone with [MW] and [RMW] during the composting duration. Furthermore, infrared spectra analysis revealed that samples from compost [EM+V] included more biodegradable components than compost with [EM]. The findings of ANOVA analysis (*p* value < 0.05) showed that the physical and chemical parameters of both [EM+V] and [EM] composts differed significantly. 

The XRD technique demonstrated the breakdown of complicated compounds into simpler components indicating cellulose degradation, and hence, based on an average crystallinity size of 69.82 nm, [RWM+EM+V] proved the accelerated maturity level of compost compared with others. Overall, the study approach suggested that a rapid composting mechanism is possible through vermi-technology-microorganisms and could be a viable option for transforming industrial raw mushroom waste into sustainable value products.

## Figures and Tables

**Table 1:** Various crop residues used in mushroom production.

Strains	Residues	References
*Agaricus bisporus*	wheat straw residues and rice straw and hulls.	[15,16]
*Pleurotus* sp.	soybean straw, coffee pulp, corn fibre, cottonseed hulls, groundnut shells, and maize straw.	[17]
*Volvallella*	paddy straw; coconut fibre, coir, and husks; cotton waste; and barley straw.	[18,19]
*Ganoderma*	sawdust, jowar leaves, sugar cane bagasse, and cottonseed hulls.	[20]

**Table 2:** Initial characteristics of the materials used for the experiment.

Parameter	Mushroom Waste (MW)	Raw Mushroom Waste (RMW)	Cow Manure
pH	7.3	7.9	9.3
TOC%	40.02	38.23	34.60
C:N ratio	39.80	38.05	45.62
MC%	55.80	54.90	58.6
N%	0.71	0.64	2.88
P%	0.19	0.22	0.32
K%	1.35	1.18	1.91

TOC: Total Organic Carbon, C:N ratio: Carbon/Nitrogen ratio, MC: Moisture Content, N: Nitrogen, P: Phosphorous, and K: Potassium.

**Table 3:** Seven averages of the cellulose peaks used to calculate the crystallinity size.

Samples	Average β = FWHM (in Radian)	Average 2θ (in Radian)	Average Crystallinity Size (nm)
MW+EM	0.4269	30.38	93.13
RWM+EM	0.4861	28.71	86.52
MW+V+EM	0.5194	25.51	72.43
RWM+V+EM	0.5376	24.16	69.82

**Table 4:** Comparative results of the mushroom waste compost.

Treatment of Mushroom Waste	Earthworm Species	Manure	Duration (Time)	C: N Ratio	References
Vermicomposting	*Lumbricus rubellus*	Cow Dung	10 Weeks	8.9 *	[1]
Vermicomposting	*Eisenia-fetida*	Cow Dung	12 Weeks	6.67 *	[11]
Vermicomposting	*Lumbricus rubellus*	Goat Manure	20 Weeks	6.39 *	[30]
Vermicomposting	*Eisenia-fetida*	Cow Dung	75 Days	11.97	[35]
Vermicomposting	*Eisenia-fetida*	Pig Dung	Four Month	10.43 *	[37]
Effective Microorganism + Vermicomposting	*Eisenia-fetida*	Cow Dung	6 Weeks	10.2 *	[PS]

* Highlight the best quality of compost with Statistically (ANOVA *p* < 0.05), [PS] Present Study.

## Data Availability

The data presented in this study are available on request from the corresponding authors.
